# Quantitative evaluation of dual-channel drug supply policy on nationally negotiated anti-tumor drugs in Xuzhou: based on interrupted time series analysis

**DOI:** 10.3389/fphar.2025.1571822

**Published:** 2025-09-03

**Authors:** Zhaohui Qin, Shuo Xu, Qi Li, Xueling Guan, Meng He, Min Zhou, Yan Xu

**Affiliations:** ^1^ The Second Clinical Medical School, Xuzhou Medical University, Xuzhou, Jiangsu, China; ^2^ School of Economics and Management, China University of Mining and Technology, Xuzhou, Jiangsu, China; ^3^ School of Management, Xuzhou Medical University, Xuzhou, Jiangsu, China; ^4^ Administrative Office, Yancheng First People’s Hospital, Yancheng, Jiangsu, China

**Keywords:** quantitative evaluation, dual-channel, anti-tumor drugs, nationally negotiated drugs, interrupted time-series analysis

## Abstract

**Introduction:**

In September 2021, the “dual-channel” supply policy for anti-tumor drugs had launched in China. This policy solved the problem of difficult and expensive access to anti-tumor drugs. This study aims to measure the impacts of the dual-channel policy on indicators of availability, cost, and reimbursement ratio.

**Methods:**

This study adopt the interrupted time series analysis method and selects data on anti-tumor drug payments from October 2020 to October 2022 in Xuzhou City for analysis.

**Results:**

After September 2021, the cost of anti-tumor drugs has increased significantly, and the number of purchases and reimbursement ratio and other indicators have increased. It is worth noting that the changes in urban and rural residents’ health insurance are more significant than those of employees’ health insurance.

**Discussion:**

The “dual-channel” supply policy has greatly improved accessibility to anti-tumor drugs. It also reduced the financial burden of disease for ordinary residents. Therefore, we need to enhance the sufficiency of policy implementation.

## Introduction

Tumors, as one of the major diseases causing disability or death, led to 4.82 million new patients and 3.21 million deaths in China in 2022. The large increase in the incidence of tumors has led to a rapid increase in the demand and price of anti-tumor drugs (Zhu et al., 2022; Eniu et al., 2019; Ruan et al., 2024). In 2021, the global expenditure on anti-tumor drugs was USD 185 billion, accounting for about 70% of the total expenditure on tumor treatment. The high expenditure is a heavy burden on patients and national medical insurance funds (Jbaily, 2022; Rahman et al., 2022). Therefore, the government needs to take measures to adjust the price and supply of tumor drugs to optimize resource allocation in the drug market (Fulone et al., 2023).

For anti-tumor drugs, different countries have adopted various approaches to control medical expenses, mainly through pricing and setting payment limits for patients. The United States government adopt a combined strategy of free pricing and government price caps. The Medicare Part D policy stipulated that the upper limit of out-of-pocket expenses for anti-cancer drugs for insured patients is $2,000 per year. The price of anti-tumor drugs in the United Kingdom is led by the government. The government conducts cost-benefit analysis to assess the value of drugs and negotiates prices based on the actual clinical effects. Doctors are free to prescribe evaluated medications, and the self-payment limit for patients using these medications is £9.35 per prescription. Switzerland uses internal reference pricing. They combining the price of the same drug in other countries, and re-evaluates it every few years. The out-of-pocket maximum cost for patients using these drugs is 10%, and the actual payment amount did not exceed 700 francs (Yuan et al., 2023).

To improve the accessibility and affordability of effective medicines for major diseases such as tumors, China has implemented a reform of national health insurance drug negotiations since 2016. With the normalization of these national drug negotiations, new and effective medicines have been continuously incorporated into the national health insurance drug list, but the new problem of “difficult access in hospitals” has emerged. Specifically, first, before the reform of the national negotiation policy, there was sufficient time for new drugs to be screened in market and clinical applications before they were included in the national health insurance drug list (Cai et al., 2022; Zhang et al., 2022a). However, after the reform, the negotiated new drugs were first included in the national health insurance drug list rather than being admitted to the hospital, which made it more difficult for hospitals to complete the access and widespread use of new drugs in a short period of time (Zhang et al., 2022a; Mingge et al., 2023). Second, after the full implementation of the DRG payment reform in 2020, the standard of medical care payments to hospitals was determined based on the recent historical prices of conventional drugs. Hospitals are hesitant to use newly listed drugs because early use of new drugs may cause certain financial losses. The above two reasons led to the difficult situation of nationally negotiated drugs entering hospitals, causing the accessibility of new drugs to not be able to meet public expectations (Xu et al., 2023; Gu et al., 2023).

To improve the accessibility of nationally negotiated drugs, China pushed forward the reform of drug catalogue management, and implemented the “dual-channel” drug supply policy (abbreviated as dual-channel policy in this article). In 2021, China's National Healthcare Security Administration (NHSA) and National Health Commission of the People's Republic of China (NHC) issued “Guiding opinions on establishing and improving the ‘Dual-Channel' management mechanism for negotiated drugs under the national health insurance” (NHSA, NHC, 2021a) and “Notice on adapting to the normalization of national medical insurance negotiations and continuously ensuring the implementation of negotiated drugs” (NHSA, NHC, 2021b). The phrase “dual-channel” refers to the two health insurance payment channels, which are designated hospitals and retail pharmacies. The “dual-channel” policy added designated retail pharmacies as a channel for purchasing and reimbursement in addition to designated hospitals, which could effectively enhance the level of supply guarantee for negotiated medicines and improved the accessibility of medicines for patients.

Due to the differences in economic conditions among provinces, the “dual-channel” policy was implemented differently in various provinces of China. In Sichuan Province, the reimbursement policy for anti-tumor drugs did not distinguish between outpatient and inpatient services. In Jiangxi Province, some clinically essential and expensive anti-tumor drugs were managed as special drugs for basic medical insurance. In Shandong Province, the costs of some anti-tumor drugs were listed separately and not included in the calculation of the drug proportion and average cost per visit of medical institutions. Anti-tumor drugs in Jiangsu Province were managed under the category of outpatient chronic diseases and conventional Class B drugs (Shen et al., 2022). Xuzhou actively implemented the “dual-channel” policy. Anti-tumor drugs with high clinical value, long service life and low substitutability are subject to classified management. This study selects the data of anti-tumor drugs in Xuzhou before and after the implementation of “dual-channel” policy. We measured the impacts of the dual-channel policy on indicators of availability, cost, and reimbursement ratio of anti-tumor drugs in Xuzhou, evaluates the effectiveness of this policy. The results can provide references for the improvement of policies in other regions.

## Data and methods

### Study area

Jiangsu Province, located in the central eastern coastal region of China, has the second largest economy in the country. Xuzhou is located in the northwestern part of Jiangsu Province and has the third largest population in the province, with a population of over 9 million in 2023, which is at the middle level among the cities in eastern China (National Health Commission of the People’s Republic of China, 2023). In October 2021, Xuzhou implemented the “dual-channel” policy. As one of the earliest cities in Jiangsu Province to implement the “dual-channel” policy, Xuzhou has achieved outstanding results and has promoted pilot experiences nationwide. Up to 2023, 74 kinds of anti-tumor drugs in Xuzhou City benefited from the “dual-channel” policy, and a total of 25 designated hospitals and designated retail pharmacies had been built.

### Sources of research data

The data were obtained from the Healthcare Insurance Data Information System of the Health Insurance Bureau of Xuzhou, which is not open to the public for patient privacy protection. The database contains detailed information on the medical treatment and registration of all urban and rural residents of Xuzhou.

The data inclusion criteria for this study are all patients in Xuzhou City who normally participate in residents’ health insurance or employees’ health insurance, using the “dual-channel” policy for reimbursement of anti-tumor drugs. According to this criteria, the data is provided by the authoritative database of the Health Insurance Bureau of Xuzhou City. The data is complete and without any omissions. There is no deletion or addition of individual cases. This study extracts data related to anti-tumor drugs among the nationally negotiated drugs in Xuzhou City from October 2020 to October 2022. The data provided for academic research is limited in scope, but it can still serve to study the initial effects of the dual-channel implementation.

## Methods

This study evaluated the impact of the “dual-channel” policy implementation from three aspects: availability, cost, and reimbursement ratio. The availability aspect included two indicators: Number of patients using anti-tumor drugs and Purchase quantity of anti-tumor drugs. The cost aspect included two indicators: Total cost of anti-tumor drugs and Per capita cost of anti-tumor drugs. The reimbursement ratio aspect included one indicator: Reimbursement ratio of anti-tumor drugs.

The study used interrupted time series analysis (ITSA) to evaluate the policy effect. ITSA is a quasi-experimental research method used to evaluate the effects of an intervention. It measures the instantaneous and trending changes of an intervention by comparing data at multiple time points before and after the intervention (Bernal et al., 2017; Wagner, 2002). ITSA has been widely used in the field of policy evaluation, mainly because of its ability to distinguish an intervention-induced impact from the original trend. It should be noted that the ITSA model assumes the absence of concurrent interventions. Xuzhou City began to implement DRG before October 2020. All the time periods selected for this study were after the implementation of DRG, and no other reforms were carried out during the same period. Moreover, high-value drugs reimbursed through the dual-channel policy are not included in the proportion of drug expenses. Therefore, the DRG implementation had no impact on the research results.

The formula of the ITSA model is as follows (Lopez et al., 2018; Chen, 2016):

$$Y=\beta_{0}+\beta_{1}T+\beta_{2}D+\beta_{3}P+\varepsilon$$

Y is a monthly outcome variable from January 2020 to October 2022, which can be the number of drug users, purchase quantity, total cost, *per capita* cost or reimbursement ratio of anti-tumor drugs. T is a continuous monthly time variable within the whole survey interval. D is a variable to distinguish between pre-policy and post-policy intervals. D is set to 0 before policy intervention and 1 after intervention. Since the dual-channel policy was implemented in Xuzhou in September 2021, this study chooses this date as the policy intervention node. P is the monthly continuous time variable after the policy intervention, i.e., P = 0 before the policy intervention and 1–12 (October 2021 to October 2022) after the policy intervention. β_0_ denotes the baseline level at T = 0. β_1_ denotes the pre-policy trend of the Y outcome. β_2_ denotes the instantaneous horizontal change after the policy. β_3_ denotes the trend change in Y compared to the pre-policy intervals. ε is the error term.

## Results

### Number of patients using anti-tumor drugs

Table 1 shows the ITSA results of the policy effect on the number of patients using anti-tumor drugs in Xuzhou. The results show that, before the implementation of the dual-channal policy, the initial number of anti-tumor drug users was 3,377.46, which increased by 225.91 per month. Specifically, the initial number of anti-tumor drug users with employees’ health insurance was 1,249.23, with an increase of 79.37 per month; the initial number of anti-tumor drug users with residents’ health insurance was 2,128.23, which increased by 146.54 per month. All of the above parameter estimates satisfy P < 0.05, indicating that the parameters of β_0_ ^1^ and β_1_ ^2^ were significant before the policy was implemented.

**TABLE 1:** ITSA results of the policy effect on the number of patients using anti-tumor drugs.

Variables	Coefficient	SE	t	P Value	95% CI
Entire population					
β_1_: baseline slope	225.91	24.58	9.19	<0.001	(174.79,277.03)
β_2_: level change after policy	−616.37	388.81	−1.59	0.128	(-1,425.2,191.95)
β_3_: slope change after policy	−15.66	50.94	−0.31	0.762	(-121.56,90.30)
β_0_: baseline level	3,377.46	229.31	14.73	<0.001	(2,900.59,3854.32)
Employee					
β_1_: baseline slope	79.37	8.97	8.85	<0.001	(60.72,98.02)
β_2_: level change after policy	−210.76	139.34	−1.51	0.145	(-500.53,79.01)
β_3_: slope change after policy	−22.30	17.77	−1.25	0.223	(-59.26,14.67)
β_0_: baseline level	1,249.23	81.52	15.32	<0.001	(1,079.70,1418.76)
Resident					
β_1_: baseline slope	146.54	16.33	8.97	<0.001	(112.57,180.50)
β_2_: level change after policy	−405.61	256.98	−1.58	0.129	(-940.04,128.81)
β_3_: slope change after policy	6.64	34.17	0.19	0.848	(-64.43,77.70)
β_0_: baseline level	2,128.23	149.98	14.19	<0.001	(1816.34,2440.12)

**FIGURE 1:**
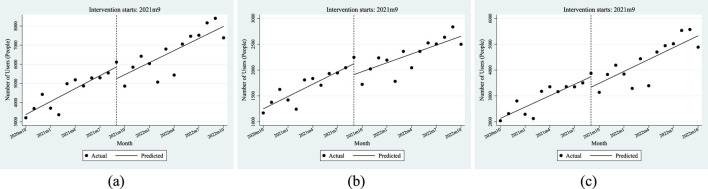
ITSA results of the policy effect on number of patients using anti-tumor drugs. **(a)** Entire population. **(b)** Employee. **(c)** Resident.

When the policy was implemented in September 2021, all relevant parameters β_2_ ^3^ were not significant. After the policy came into effect in September 2021, the total number of anti-tumor drug purchases increased each month. But all relevant parameters β_3_ ^4^ in Table 1 do not pass the parameter significance test. The above results, combined with the trend presented in Figure 1, show that this policy had no significant impact on the number of patients using anti-tumor drugs in Xuzhou.

### Purchase quantity of anti-tumor drugs

Table 2 shows the dual-channel policy’s influences on the purchase quantity of anti-tumor drugs in Xuzhou City. Before its implementation, the initial purchase quantity was 8,958, which increased by 558 per month. Specifically, the initial quantity of employees with health insurance was 3,299, with an increase of 189 per month; the initial quantity of residents with health insurance was 5,659, which increased by 369 per month. All of the above parameter estimates satisfy P < 0.05, indicating that the parameters of β_1_ and β_0_ were significant before the dual-channel policy was implemented.

**TABLE 2:** ITSA results of the policy effect on the purchase quantity of anti-tumor drugs.

Total	Coefficient	SE	t	P Value	95% CI
Entire population					
β_1_: baseline slope	558.00	76.65	7.28	<0.001	(398.41,727.23)
β_2_: level change after policy	−760.00	1,125.92	−0.68	0.507	(-3,101.47,1581.47)
β_3_: slope change after policy	434.00	199.06	2.18	0.041	(20.15,848.09)
β_0_: baseline level	8,958.00	705.29	12.70	<0.001	(7,490.82,10,424.27)
Employee					
β_1_: baseline slope	189.00	35.51	5.32	<0.001	(114.95,262.67)
β_2_: level change after policy	571.00	352.94	1.62	0.121	(-163.17,1304.80)
β_3_: slope change after policy	77.00	57.88	1.33	0.196	(-43.15,197.57)
β_0_: baseline level	3,299.00	301.64	10.93	<0.001	(2,671.02,3925.62)
Resident					
β_1_: baseline slope	369.00	44.51	8.29	<0.001	(276.45,461.57)
β_2_: level change after policy	−1,331.00	1,032.95	−1.29	0.212	(-3,478.94,817.32)
β_3_: slope change after policy	357.00	171.52	2.08	0.050	(0.22,713.61)
β_0_: baseline level	5,659.00	419.58	13.49	<0.001	(4,786.66,6531.79)

When the policy was implemented in September 2021, all relevant parameters β2 were not significant. After the policy came into effect, the total number of anti-tumor drug purchases increased each month. The purchase quantity increased by an additional 434 units per month, with the residents’ health insurance (357) being the main growth point, and the corresponding parameter estimates were significant. The employees’ health insurance (77) was not significant.

**FIGURE 2:**
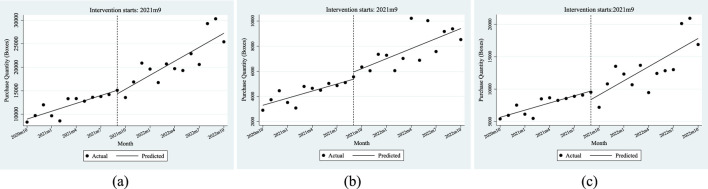
ITSA results of the policy effect on the purchase quantity of anti-tumor drugs. **(a)** Entire population. **(b)** Employee. **(c)** Resident.

The above results and the trend presented in Figure 2 show that the demand for anti-tumor drugs rises every month. Compared with employees, the policy has a more significant impact on the purchase of anti-tumor drugs by residents.

### Total cost of anti-tumor drugs

Table 3 shows the ITSA results of the policy influences on the total cost of anti-tumor drugs in Xuzhou City. The results indicate that, before the implementation of the dual-channel policy, the initial level of the total cost of anti-tumor drugs was CNY 28.41 million, which increased by CNY 0.67 million per month. Specifically, the initial level of the total cost of employees’ health insurance was CNY 11.04 million, with an increase of CNY 0.2 million per month. The initial level of the cost of residents’ health insurance was CNY 17.37 million, which increased by CNY 0.47 million per month. All of the above parameter estimates satisfy P < 0.05, indicating that the parameters of β_0_ and β_1_ were significant before the dual-channel policy came into effect.

**TABLE 3:** ITSA results of the policy effect on the total cost of anti-tumor drugs.

Variables	Coefficient	SE	t	P Value	95% CI
Entire population					
β_1_: baseline slope	0.67	0.27	2.49	0.021	(11.10, 1.24)
β_2_: level change after policy	0.17	2.75	0.06	0.950	(-5.55, 5.90)
β_3_: slope change after policy	0.90	0.40	2.23	0.037	(0.06, 1.73)
β_0_: baseline level	28.41	2.23	12.72	<0.001	(23.76,33.05)
Employee					
β_1_: baseline slope	0.20	0.01	2.01	0.057	(-0.07, 0.40)
β_2_: level change after policy	0.10	0.99	0.10	0.919	(-1.95, 2.15)
β_3_: slope change after policy	0.18	0.15	1.24	0.228	(-0.12, 0.49)
β_0_: baseline level	11.04	0.79	13.88	<0.001	(9.38, 12.69)
Resident					
β_1_: baseline slope	0.47	0.17	2.72	0.013	(0.11 0.84)
β_2_: level change after policy	0.07	1.86	0.04	0.969	(-3.81, 3.95)
β_3_: slope change after policy	0.72	0.27	2.70	0.013	(0.16, 1.27)
β_0_: baseline level	17.37	1.45	11.99	<0.001	(14.36, 20.38)

When the policy was implemented in September 2021, all relevant parameters β2 were not significant. After the policy came into effect, the monthly increase in the total cost of anti-tumor drugs was relatively increased by CNY 0.9 million, with the residents’ health insurance (0.72) being the main growth point, and the corresponding parameter estimates were significant. The employees’ health insurance (0.18) was not significant.

**FIGURE 3:**
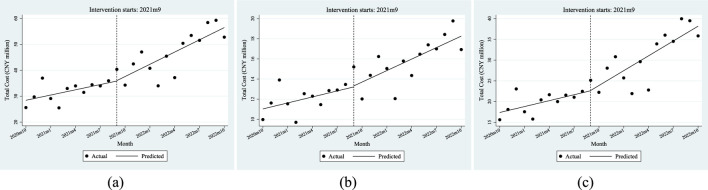
ITSA results of the policy effect on the total cost of anti-tumor drugs. **(a)** Entire population. **(b)** Employee. **(c)** Resident.

The ITSA results indicated in Figure 3 show that the total cost of anti-tumor drugs grew faster after the policy was implemented. Furthermore, the policy has a greater impact on the total cost of residents than employees.

### Per capita cost of anti-tumor drugs

Table 4 shows the ITSA results of the policy influences on the *per capita* cost of anti-tumor drugs in Xuzhou City. The results show that, before the implementation of the dual-channel policy, the initial level of the *per capita* cost of anti-tumor drugs was CNY 8228.76, which decreased by CNY 212.8 per month. Specifically, the initial level of the *per capita* cost of anti-tumor drugs covered by employees’ health insurance was CNY 8635.85, with a decrease of CNY 241.66 per month; the initial level of the *per capita* cost of anti-tumor drugs covered by residents’ health insurance was CNY 7990.36, which decreased by CNY 195.54 per month. All of the above parameter estimates satisfy P < 0.05, indicating that the parameters of β_0_ and β_1_ were significant before the dual-channel policy came into effect.

**TABLE 4:** ITSA results of the policy effect on the *per capita* cost of anti-tumor drugs.

Variables	Coefficient	SE	t	P Value	95% CI
Entire population					
β_1_: baseline slope	−212.80	29.19	−7.29	<0.001	(-273.50, −152.10)
β_2_: level change after policy	997.71	246.43	4.05	0.001	(485.23, 1,510.19)
β_3_: slope change after policy	226.97	32.89	6.90	<0.001	(158.57, 295.37)
β_0_: baseline level	8,228.76	188.47	43.66	<0.001	(7,836.81, 8,620.70)
Employee					
β_1_: baseline slope	−241.66	24.97	−9.68	<0.001	(-293.60, −189.72)
β_2_: level change after policy	988.41	215.47	4.59	0.001	(540.31, 1,436.51)
β_3_: slope change after policy	234.60	26.99	8.69	<0.001	(178.47, 290.74)
β_0_: baseline level	8,635.85	142.16	60.75	<0.001	(8,340.22, 8,931.48)
Resident					
β_1_: baseline slope	−195.54	32.21	−6.07	0.001	(-262.53, −128.55)
β_2_: level change after policy	1,001.00	272.02	3.68	0.001	(435.31, 1,566.69)
β_3_: slope change after policy	220.74	37.50	5.89	<0.001	(142.75, 298.73)
β_0_: baseline level	7,990.36	217.16	36.79	<0.001	(7,538.74, 8,441.98)

From the perspective of instantaneous change, after the policy landed in September 2021, the *per capita* cost of anti-tumor drugs instantaneously increased by CNY 997.71, of which the *per capita* cost of employees’ health insurance increased by CNY 988.41, and the *per capita* cost of residents’ health insurance increased by CNY 1001. From the perspective of the trend change after the policy came into effect, the monthly increase in the *per capita* cost of anti-tumor drugs was relatively increased by CNY 226.97, of which the cost of people with employees’ health insurance increased by CNY 234.6, and the cost of residents’ health insurance increased by an additional CNY 220.74 per month. All of the above parameter estimates satisfy P < 0.05, indicating that the parameters of β_2_ and β_3_ were significant when the dual-channel policy was implemented.

**FIGURE 4:**
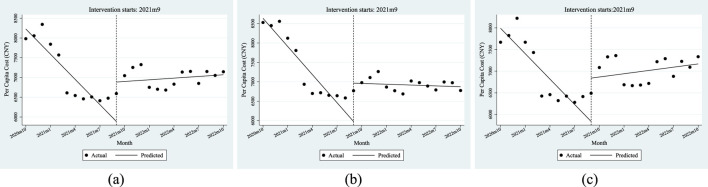
ITSA results of the policy effect on the *per capita* cost of anti-tumor drugs. **(a)** Entire population. **(b)** Employee. **(c)** Resident.

Combined with the trend seen in Figure 4, the above results show that the *per capita* cost of anti-tumor drugs changed from a downward trend before the dual-channel policy came into effect to a monthly increase after the policy implementation.

### Reimbursement ratio of anti-tumor drugs

Table 5 shows the ITSA results of the policy influences on the reimbursement ratio of anti-tumor drugs in Xuzhou City. The results show that, before the implementation of the dual-channel policy, the initial level of reimbursement ratio of anti-tumor drugs was 51.82%, which decreased by 0.01% per month. Specifically, the initial level of reimbursement ratio of anti-tumor drugs covered by employees’ health insurance was 54.81%, with an increase of 0.01% per month; the initial level of reimbursement ratio of anti-tumor drugs covered by residents’ health insurance was 49.92%, which decreased by 0.01% per month. Most of the above parameter estimates satisfy P < 0.05 except β_1_ of the residents’ reimbursement ratio of anti-tumor drugs, indicating that most of the parameters of β_0_ and β_1_ were significant before the dual-channel policy landed.

**TABLE 5:** ITSA results of the policy effect on the reimbursement ratio of anti-tumor drugs.

Variables	Coefficient	SE	t	P Value	95% CI
Entire population					
β_1_: baseline slope	−0.01	0.00	−2.18	0.041	(-0.02, 0.00)
β_2_: level change after policy	7.41	2.24	3.30	0.003	(2.74, 12.07)
β_3_: slope change after policy	0.17	0.25	0.66	0.515	(-0.36, 0.69)
β_0_: baseline level	51.82	0.03	2008.46	0.000	(51.77, 51.87)
Employee					
β_1_: baseline slope	0.01	0.00	2.30	0.032	(0.00, 0.01)
β_2_: level change after policy	10.00	3.03	3.30	0.003	(3.70, 16.30)
β_3_: slope change after policy	0.28	0.34	0.83	0.414	(-0.42, 0.99)
β_0_: baseline level	54.81	0.02	2,874.72	0.000	(54.77, 54.85)
Resident					
β_1_: baseline slope	−0.01	0.00	−1.35	0.190	(-0.01, 0.00)
β_2_: level change after policy	5.95	1.86	3.19	0.004	(2.08, 9.82)
β_3_: slope change after policy	0.14	0.21	0.66	0.519	(-0.30, 0.57)
β_0_: baseline level	49.92	0.02	2,333.30	0.000	(49.88, 49.96)

From the perspective of instantaneous change, when the policy came into effect, the reimbursement ratio of anti-tumor drugs instantaneously increased by 7.41%, of which the number of people with employees’ health insurance increased by 10.00%, and the number of people with residents’ health insurance increased by 5.95%. From the perspective of the trend change after the policy was implemented, the monthly increase in the reimbursement ratio of anti-tumor drugs was relatively increased by 0.17%, of which the number of people with employees’ health insurance increased by 0.28%, and those with residents’ health insurance increased by an additional 0.14% per month. All of the above parameter estimates of β_2_ satisfy P < 0.05. After the policy came into effect, all relevant parameters β3 were not significant.

**FIGURE 5:**
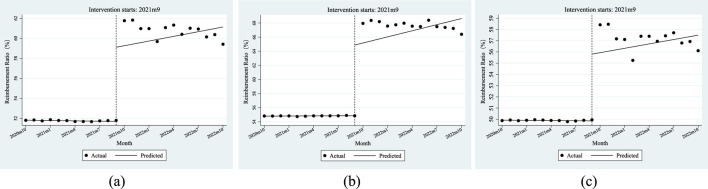
ITSA results of the policy effect on the reimbursement ratio of anti-tumor drugs. **(a)** Entire population. **(b)** Employee. **(c)** Resident.

Figure 5 shows that the reimbursement ratio of anti-tumor drugs had a relatively stable change trend before and after the dual-channel policy came into effect but had a significant improvement at the moment of the implementation of the policy.

## Discussion

This article provides a comprehensive overview of the changes in the number of patients and quantity of drugs, total and *per capita* costs, and reimbursement ratio for patients who purchased nationally negotiated anti-tumor drugs after the implementation of the dual-channel policy in Xuzhou. We evaluate the influence of the policy from three perspectives: availability, cost and reimbursement ratio.

### Availability perspective

From the perspective of the number of drugs covered, the number of people using anti-tumor drugs rose before the policy intervention in Xuzhou. Besides, whether they were participating in which kind of medical insurance, the statistical significance of the changing trend of the number of paitients using anti-tumor drugs after the implementation of the policy was not obvious. Because of the prevalence of tumor diseases, the number of users for anti-tumor drugs was gradually increasing in Xuzhou, and the implementation of the “dual-channel” policy made it more convenient for people to obtain anti-tumor drugs (Ostrer and Gross, 2022; Zou et al., 2023). However, as the implementation time of this policy is relatively short in Xuzhou, it still needs to be strengthened in terms of the sufficiency of policy implementation and public acceptance. Therefore, the number of users did not show a statistically significant increase.

From the perspective of the trend of changes in purchase quantity, the purchase quantity of drugs significantly increased before and after the policy came into effect in Xuzhou. The increase in purchase quantity after the policy intervention was greater than that before in the urban and rural residents’ health insurance. The “dual-channel” policy established a fund settlement channel between the medical insurance office and certified retail pharmacies, giving full play to the advantages of pharmacies’ wide distribution and flexible services, which had increased the prevalence of anti-tumor drugs and played a positive role in alleviating the “difficulty in landing” the drugs (Zhang et al., 2022b; Xu et al., 2022).

### Cost perspective

From the perspective of total cost, after the implementation of the policy, the total cost of anti-tumor drugs has an obvious upward trend in Xuzhou, and the monthly growth rate of resident medical insurance is more significant than that of employee medical insurance. The reason is that employee medical insurance is essentially perfect due to its welfare policy (Li et al., 2019; Zhang et al., 2023c; Ren et al., 2022), which is scarcely affected by the policy, so the changing trend of the total cost is not obvious. The scope of drug reimbursement of residents’ medical insurance is smaller than that of employees’ medical insurance. The “dual-channel” policy ensures the accessibility of drugs to the general public and greatly improves the welfare of resident medical insurance in Xuzhou, thus driving the number of users and the purchase quantity of anti-tumor drugs, resulting in an increase in the total cost of anti-tumor drugs.

From the perspective of *per capita* cost, the *per capita* cost of anti-tumor drugs decreased before the policy intervention in Xuzhou. After the policy intervention, the *per capita* cost of employee medical insurance decreased significantly, and the average cost of resident medical insurance changed from decreasing to slightly increasing. It can be seen that the implementation of “two-channel” policy in Xuzhou ensures the channels for ordinary residents to obtain anti-tumor drugs, pushes up the purchased quantity by users, and leads to a rise in *per capita* cost. This may be because, before the “double channel” policy was implemented, most of the drugs for tumor patients could only be reimbursed in the hospital outpatient department. In the context of DRG reform, the total settlement of medical insurance in medical institutions decreases, and hospitals will control the cost when supplying anti-tumor drugs (Li et al., 2023; Baltin et al., 2023). The “dual channel” policy clarifies the payment scope and standard of anti-tumor drugs, and reduces the pressure on medical institutions’ drug equipment and medical services. Drugs are deployed, supplied and used outside the hospital, and the problem of “difficult access in hospitals” has been greatly alleviated (Liu et al., 2022; Sun et al., 2022).

### Reimbursement ratio perspective

From the perspective of reimbursement level analysis, the actual reimbursement ratio of anti-tumor drugs was stable at a low level before the policy intervention, and increased significantly after it in Xuzhou. The reason is that, in September 2021, the policy reimbursement rate of anti-tumor drugs in Xuzhou increased from 55% to 70% in employees’ health insurance, and 50%–60% in residents’ health insurance. The change in the policy reimbursement rate led to an instant increase in the actual reimbursement rate. The relatively short period of policy implementation may have contributed to the large fluctuations in actual reimbursement rates and insignificant parameter estimates.

### Recommendations and outlook

The “dual-channel” policy significantly increased the accessibility of anti-tumor drugs and reduced the medical burden of patients, but there are also some shortcomings (Rawson, 2022; Religioni and Pakulska, 2020).

First, the implementation of the “dual-channel” policy brought about a continuous increase in the number of drug users and the total amount of drugs used, and it is necessary to ensure the stability of the supply chain of anti-tumor drugs. However, the current mechanism of China’s “dual-channel” drug policy could be improved, and some hospitals are reluctant to equip themselves with the relevant drugs. Moreover, the continuous shortage of anti-tumor drugs made it urgent to accelerate the supply of anti-tumor drugs (Zhou et al., 2024).

Second, the rationality of doctors’ medication also needs to be strengthened. Anti-tumor drugs are relatively expensive and have complex indications, the “dual-channel” policy has expanded the application of anti-tumor drugs, which have higher requirements for doctors’ medication level. Hospitals should strengthen the supervision of the responsible physician’s use of medication to further regulate the use of medication (Fundytus et al., 2020).

### Limitations of the present study

1. This study only collected data indicators on anti-tumor drugs from 2020 to 2022; observations on the cost and number of purchasers of anti-tumor drugs before 2020 are limited. On the condition that further authorization for accessing the data from the health insurance bureau is obtained, the subsequent research can further extend the time frame to study the longer-term effects of the policy.
2. This study may have a policy change trend of being slow at first and then urgent. The relatively short post-policy observation period cannot accurately describe the change trend after policy intervention and may misestimate the long-term impact of the policy.
3. The implementation of the dual-channel policy has economic disparities and heterogeneity. Xuzhou’s good implementation results cannot be unconditionally extrapolated to other regions of China, limiting the general applicability of the findings.
4. The indicators selected in this study show some consistency, indicating that there are internal mixed factors.

## Conclusion

This study adopted the interrupted time series analysis method to analyze the influence of the “dual-channel” supply policy in Xuzhou. The results showed that after the implementation of the policy, the indicators of the anti-tumor drugs had a great upward trend from the perspectives of availability, cost and reimbursement ratio in Xuzhou. It demonstrated that this policy significantly increased the accessibility of anti-tumor drugs and reduced the healthcare burden of patients, resulting in a significant direct intervention impact in Xuzhou. This finding provided substantial positive evidence of the “dual-channel” policy in facilitating the widespread dissemination of nationally negotiated drugs and relieving patients’ financial stress. It is worth noting that, compared with employees’ medical insurance, the changing trend of urban and rural residents’ medical insurance is more significant. The heterogeneity of the “dual-channel” policy in residents’ and employees’ medical insurance reflects its remarkable effect on protecting the welfare of patients with residents’ medical insurance. It should be noted that Xuzhou has a relatively high economic level and implemented the “dual-channel” policy relatively early. For other cities, they should, based on their own actual conditions, partially refer to rather than completely draw on the experience of Xuzhou.

From the perspective of the implementation effect of the policy in Xuzhou City, the dual-channel policy has expanded the medical insurance coverage of anti-tumor drugs. It also improved the convenience for patients to use anti-tumor drugs. However, China relies on national negotiations to expand the scope of medical insurance reimbursement at present. The access speed of some innovative drugs is relatively slow, and a few high-priced drugs still require patients to pay for them themselves. In the future, the government should not only adjust the drug supply policy according to the changes in the market and medical demands, but also continue to optimize the “dual-channel” drug compensation mechanism, and introduce advanced tumor drugs and technologies promptly, such as bispecific T cell engager and CRISPR gene editing (Gong et al., 2024; Xue et al., 2024). It is necessary to strengthen the cooperation among the medical insurance department, hospitals, universities and pharmaceutical companies, so that patients can obtain more effective drugs and benefit from them as soon as possible.

## Data Availability

The data that support the findings of this study are available from the corresponding author upon reasonable request and with permission of Xuzhou Medical Insurance Bureau. Requests to access the datasets should be directed to ZQ, qzh@xzhmu.edu.cn.

## Footnotes

1 β_0_ denotes the baseline level at T = 0

2 β_1_ denotes the pre-policy trend of the Y outcome

3 β_2_ denotes the instantaneous horizontal change after the policy

4 β_3_ denotes the trend change in Y compared to the pre-policy intervals
